# The Effect of Gut Microbiota-Targeted Interventions on Neuroinflammation and Motor Function in Parkinson’s Disease Animal Models—A Systematic Review

**DOI:** 10.3390/cimb46050244

**Published:** 2024-04-26

**Authors:** Paul-Ștefan Panaitescu, Vlad Răzniceanu, Ștefania-Maria Mocrei-Rebrean, Vlad Sever Neculicioiu, Hanna-Maria Dragoș, Carmen Costache, Gabriela Adriana Filip

**Affiliations:** 1 Department of Physiology, Iuliu Hatieganu University of Medicine and Pharmacy, 400006 Cluj-Napoca, Romania; panaitescu.paul.stefan@elearn.umfcluj.ro (P.-Ș.P.); mocrei.rebrean.stef.maria@elearn.umfcluj.ro (Ș.-M.M.-R.); 2 Department of Microbiology, Iuliu Hatieganu University of Medicine and Pharmacy, 400012 Cluj-Napoca, Romania; neculicioiu.vlad.sever@elearn.umfcluj.ro (V.S.N.);; 3 Department of Neurology, Iuliu Hatieganu University of Medicine and Pharmacy, 400012 Cluj-Napoca, Romania

**Keywords:** neurodegenerative disorders, gut–brain axis, inflammation, fecal microbiota transplant, prebiotics, probiotics, synbiotics, antibiotics, microglial activation, microbiome

## Abstract

Gut microbiome-targeted interventions such as fecal transplant, prebiotics, probiotics, synbiotics, and antibiotic gut depletion are speculated to be of potential use in delaying the onset and progression of Parkinson’s disease by rebalancing the gut microbiome in the context of the gut–brain axis. Our study aims to organize recent findings regarding these interventions in Parkinson’s disease animal models to identify how they affect neuroinflammation and motor outcomes. A systematic literature search was applied in PubMed, Web of Science, Embase, and SCOPUS for gut microbiome-targeted non-dietary interventions. Studies that investigated gut-targeted interventions by using in vivo murine PD models to follow dopaminergic cell loss, motor tests, and neuroinflammatory markers as outcomes were considered to be eligible. A total of 1335 studies were identified in the databases, out of which 29 were found to be eligible. A narrative systematization of the resulting data was performed, and the effect direction for the outcomes was represented. Quality assessment using the SYRCLE risk of bias tool was also performed. Out of the 29 eligible studies, we found that a significant majority report that the intervention reduced the dopaminergic cell loss (82.76%, 95% CI [64.23%, 94.15%]) produced by the induction of the disease model. Also, most studies reported a reduction in microglial (87.5%, 95% CI [61.65%, 98.45%]) and astrocytic activation (84,62%, 95% CI [54.55%, 98.08%]) caused by the induction of the disease model. These results were also mirrored in the majority (96.4% 95% CI [81.65%, 99.91%]) of the studies reporting an increase in performance in behavioral motor tests. A significant limitation of the study was that insufficient information was found in the studies to assess specific causes of the risk of bias. These results show that non-dietary gut microbiome-targeted interventions can improve neuroinflammatory and motor outcomes in acute Parkinson’s disease animal models. Further studies are needed to clarify if these benefits transfer to the long-term pathogenesis of the disease, which is not yet fully understood. The study had no funding source, and the protocol was registered in the PROSPERO database with the ID number CRD42023461495.

## 1. Introduction

### 1.1. Parkinson’s Disease—Clinical and Pathophysiological Characterization

Six million cases of Parkinson’s disease (PD) have been recorded between 1990 and 2015, and this number is expected to double by 2040 as a result of population aging and growth [1,2]. Over one million deaths due to PD have been recorded between 1994 and 2019 [3], and mortality increases with disease duration regardless of levodopa therapy [4], making it a necessity to develop disease-modifying therapies for PD [5]. PD is a common neurodegenerative disease with disabling motor clinical signs such as rigidity, tremors, and gait disturbances accompanied by non-motor symptoms like cognitive decline and constipation [6]. Its pathophysiological traits are dopaminergic neuron loss in the substantia nigra pars compacta (SNpc) [7] and the intraneuronal accumulation of α-synuclein (α-syn) aggregates or Lewy bodies throughout the central and peripheral nervous systems [8,9,10].

### 1.2. The Gut–Brain Axis and Its Role in PD Pathogenesis

The observation that Lewy bodies first appear in the gut and that gastrointestinal symptoms precede motor symptoms has led to the hypothesis that PD may originate in the gut as a multisystemic pathology [11]. This could be explained through the gut–brain axis, a bidirectional communication system between the brain and the gut involving microbiota, and its relationship with immune, endocrine, metabolic, and neural pathways [12]. α-Syn has been shown to accumulate bidirectionally along this axis in the brain and the enteric nervous system (ENS) [13]. Braak’s hypothesis proposes a dual hit model of sporadic body-first PD wherein a pathogen triggers the gastrointestinal tract (GIT) and olfactory nerve, allowing for subsequent retrograde α-syn transmission via the vagus and olfactory bulb to the SNpc [14,15]. Indeed, in prodromal PD, increased intestinal permeability or ‘leaky gut’ consequent to mucous injury and dysbiosis contributes to local and systemic inflammation, gut α-syn aggregation, blood-brain barrier (BBB) permeabilization, and microglial activation [12,14,15,16]. Microbiota involvement seems to be mediated by the CD4+ T cell response, which leads to local IFN-γ, IL-17, TNF, and IL-5 secretion [15]. By contrast, the brain-derived neurotrophic factor (BDNF) promotes intestinal tight junction integrity and is usually upregulated in colonic inflammation [17]. In PD, low BDNF decreases gut zonula occludens-1 (ZO-1), occludin, and claudin-1 expressions leading to tight junction dysfunction [18,19].

### 1.3. The Role of the Gut Microbiota in PD Pathogenesis

The involvement of the gut microbiota in PD pathogenesis is supported by correlations between altered polymicrobial clusters and disease symptoms [20]. A healthy gut microbiota includes high levels of *Bifidobacteria* and *Bacteroidetes* and low levels of *Bacillota* (previously *Firmicutes*) and *Pseumonadota* (previously *Proteobacteria*) [21]. The stability of this composition is subject to change with age, favoring PD onset in genetically predisposed individuals [22]. The PD gut microbiota has been characterized by chronic *H. pylori* infection, an increase in the *Ralstonia*, *Eubacterium*, *Lactobacillus*, *Akkermansia*, and *Bifidobacterium* genera as well as the *Enterobacteriaceae* family, and a relative decrease in the *Bacteroidetes phylum, Prevotellaceae* family, and *Faecalibacterium*, *Blautia*, and *Coprococcus* genera [12,14,23]. This is relevant because dysbiosis can lower intestinal barrier integrity and promote intestinal inflammation [21].

### 1.4. CNS and GIT Inflammatory Biomarkers in PD

Intestinal inflammation in PD can be characterized through serum, local, and fecal markers. The elevated serum markers correlated with colonic inflammation are interleukin-1 β (IL-1β), interleukin-6 (IL-6), interleukin-17 (IL-17), and Tumor Necrosis Factor alpha (TNF-α) [24,25,26]. Meaningful but non-disease-specific [27] fecal markers for intestinal dysfunction and inflammation are calprotectin and lactoferrin [26,28,29], vascular endothelial growth factor receptor 1, interleukin-1 α (IL-1α), IL-1β and interleukin-8 (IL-8, CXCL8) [30]. The intestinal barrier permeability markers zonulin and alpha-1-antitrypsin are also elevated in PD [27,31]. Highly expressed local inflammatory markers include inducible nitric oxide synthase (iNOS), TNF-α, IL-1β, IL-8, IL-6, C-reactive protein (CRP), and the inflammation-related proteins toll-like receptor 4 (TLR4), myeloid differentiation primary response protein 88 (MyD88), and nuclear factor-κB (NF-κB) [19,24,32]. PD is further characterized by both peripheral and CNS inflammatory responses [33]. The most widely investigated inflammatory markers elevated both in the CSF and serum are CRP, TNF-α, IL-6, and IL-1β [34], with the first two being strongly correlated with PD severity [35,36].

### 1.5. Non-Dietary Gut Microbiota-Targeted Interventions

Symptomatic PD management is currently based on dopaminergic therapies as well as monoamine oxidase inhibitors [37]. However, the need for disease-modifying approaches prompts further exploration of alternatives like immunotherapy and gene therapy [38]. In light of the impact of dysbiosis on PD progression, microbiota-targeted therapies such as probiotics, prebiotics, synbiotics, fecal microbiota transplantation [23,39], and non-absorbable antibiotics meant to restructure the gut microflora are promising research avenues [40,41,42]. Fecal microbiota transplantation (FMT) implies the curative translocation of gut microbiota from a healthy individual into a recipient with dysbiosis [43]. The applications to neuropsychiatric disorders like autism spectrum disorder or epilepsy are currently being explored in both animals and humans, but the supporting evidence is limited [44]. While various in vivo experiments have yielded exciting results for motor manifestations and neuroinflammation in PD [45,46,47], more extensive randomized controlled trials (RCTs) and mechanistic investigations are necessary [15,43]. Probiotics are live microorganisms that, when ingested in appropriate amounts, positively affect resident intestinal microbiota and improve the symptomatology of a wide range of chronic diseases, especially GIT ones [48,49,50]. Preliminary human studies have shown improvements in intestinal symptoms [51,52,53], depression [54], and motor and metabolic markers [55]. Murine data support their anti-inflammatory potential in PD therapy [23,56]. Prebiotics represent non-digestible substrates that the resident microflora can utilize to relieve constipation and improve immune regulation [49]. Synbiotics merge probiotics and prebiotics and can either act synergistically by having the supplemented bacteria consume the prebiotics, or in a complementary manner by having the two components exert their functions independently [49,57,58].

### 1.6. Clinical Translation of Rodent Studies on Microbiota-Targeted Therapies in PD

Interventional clinical studies are required to confirm previous findings on murine gut microbiota involvement in PD pathogenesis [59,60], and the precise molecular mechanisms involved remain to be explored [61]. The long-term safety of microbiota-targeted therapies in PD has yet to be thoroughly evaluated. While preliminary studies regarding the impact of probiotics on GI and motor symptoms have rendered promising results [62,63,64], long-term efficacy and safety studies [65], levodopa interaction risk evaluations [15], as well as therapeutic dose identification [62,66] are necessary. In comparison, FMT studies are more heterogeneous methodologically despite the limited data available on PD models [44]; some short-term studies attest to its effectiveness in treating gastrointestinal dysfunction and improving motor symptoms [45,46,67,68]. Human studies on prebiotic and synbiotic interventions in neurodegenerative diseases are even more scarce [69,70,71]

Finally, solid preclinical evidence must precede the design of large RCTs on the efficacy and safety of these interventions. Hence, preclinical PD research disposes of a wide variety of induction methods and prospective therapies with advantages and limitations in replicating PD pathogenesis. Numerous and methodologically diverse murine studies on microbiota-oriented interventions for PD have been published in recent years. However, their heterogeneity makes drawing a unified set of conclusions to help guide later translational research challenging. This systematic review aims to answer the question: “How are neuroinflammation and motor outcomes influenced by gut microbiota-targeted interventions in Parkinson’s disease murine models?”

## 2. Materials and Methods

### 2.1. Search Strategy and Protocol Registration

Prisma guidelines [72] were used for systematic searching, screening, and data extraction. The Syrcle risk of bias tool [73] was used to assess the risk of bias in the studies. A systematic review without meta-analysis was performed for data synthesis due to the high variability in methodologies and measured outcomes. Preliminary searches were performed with keywords to assess the feasibility of a systematic review on the subject. Enough research on the subject was identified to justify such an endeavor. As a result, the systematic search was performed on 1 October 2023 in four databases on the same day: PubMed, Web of Science, Scopus, and Embase. Retrieved studies in English were considered regardless of publication date; no other limits, restrictions, or filters were applied. No citation searching was performed, and no supplementary data was sought by contacting the authors. The protocol of the study was registered and reviewed by PROSPERO, and necessary changes were addressed. The resulting document can be found in the database with the ID CRD42023461495 [74].

The detailed search strategies were as follows:

- PubMed: (“parkinsonian disorders”[MeSH Terms] OR (“parkinsonian”[All Fields] AND “disorders”[All Fields]) OR “parkinsonian disorders”[All Fields]) AND ((“fecal microbiota transplantation”[MeSH Terms] OR (“fecal”[All Fields] AND “microbiota”[All Fields] AND “transplantation”[All Fields]) OR “fecal microbiota transplantation”[All Fields]) OR (“probiotics"[MeSH Terms] OR “probiotics”[All Fields]) OR (“prebiotics”[MeSH Terms] OR “prebiotics”[All Fields]) OR (“anti-bacterial agents”[All Fields] OR “anti-bacterial agents”[MeSH Terms] OR (“anti-bacterial”[All Fields] AND “agents"[All Fields]) OR “anti-bacterial agents”[All Fields] OR “antibiotics”[All Fields]));
- SCOPUS: TITLE-ABS-KEY (neuroinflammation AND (Parkinson AND disease) AND ((fecal AND microbiota AND transplantation) OR probiotics OR prebiotics OR (anti-bacterial AND agents) OR antibiotics));
- Embase: (parkinsonism)/br AND ((‘fecal microbiota transplantation’)/br) OR ((‘probiotic agent’):ti) OR ((‘prebiotic agent’):ti) OR ((‘antiinfective agent’):ti) OR ((‘antibiotic agent’):ti);
- Web of Science: Parkinson’s AND (fecal microbiota transplantation OR (fecal AND microbiota AND transplantation) OR probiotics OR prebiotics OR anti-bacterial agents OR (anti-bacterial AND agents) OR antibiotics).

### 2.2. PRISMA Guidelines

**Figure 1:**
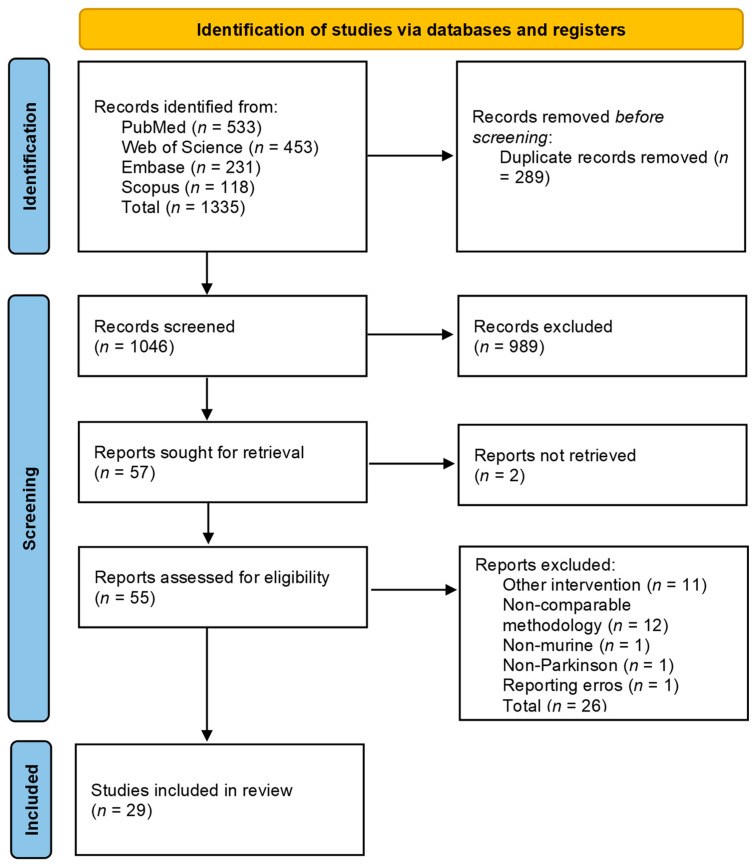
Prisma flowchart [72].

The use of PRISMA guidelines can be seen summarized in the PRISMA flowchart (Figure 1). The Rayyan tool [75] was used to automatically identify the duplicates, the elimination of which was performed only after a reviewer manually confirmed the potential duplicate. Two researchers (Ș-M. M-R. and V.R.) performed title and abstract screening, and the conflict was resolved after a third researcher (P.P.) mediated a consensus. All the articles that did not meet the inclusion criteria (Table 1) were excluded. If a study included an in vitro or a human study as well as an in vivo murine branch, the branch of interest for the study was included while the other was not. The same methodology was applied if other interventions were also used. The resulting articles were sought for retrieval, and the retrieved articles were assessed for eligibility by two researchers (Ș-M. M-R. and V.R.). Differences were solved through consensus by a third researcher (P.P.). The PRISMA Checklist, PRISMA Abstracts Checklist, and PRISMA-S Checklist can be found in Supplementary Data (File S1–S3).

For the eligible studies, data extraction was performed by two researchers (P.P. and V.N.) using the Excel data extraction template summarized in Table 2. Differences in data extraction were solved by expert oversight (A.F.). Only the groups with relevant interventions and controls were considered. The corresponding significance level was included where the outcomes were reported, with non-significant findings reported.

### 2.3. SYRCLE Risk of Bias

**Figure 2:**
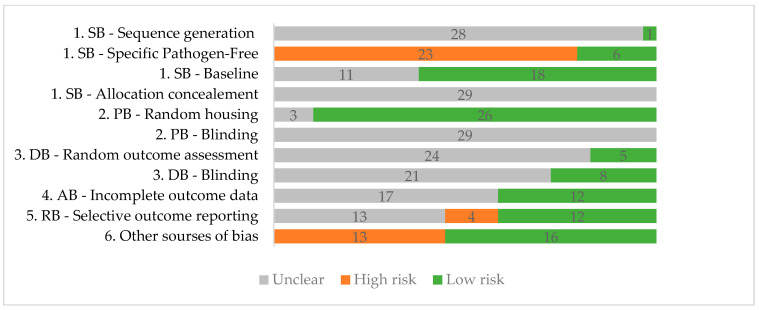
Risk of bias Syrcle [73]. SB—selection bias; PB—performance bias; DB—detection bias; AB—attrition bias; RB—reporting bias.

The risk of bias was assessed using a modified SYRCLE risk of bias tool [73] by two researchers, with differences being resolved through consensus by a third researcher. For specific microbiome-targeted interventions, we consider that a possible risk of bias that needs to be addressed is the potential differences in the microbiomes induced either by living conditions or by pathogen exposure. As such, we included a new item named “Specific pathogen-free” (SPF) for the selection bias, referring to the special conditions in which pathogen-free animals are bred and raised. Where the SPF status of the animals was mentioned, a low risk of bias was assigned. If the animals were not SPF, a high risk of bias was assigned. We do not consider that this category would fully mitigate possible discrepancies in microbiome composition between groups. As such, a pretest-posttest design for the relative abundance analysis was also considered a feasible way to lower the potential bias. For the other biases, administering the intervention ad libitum in the drinking water was considered a high risk of bias. For this method of administration, housing single animals per cage with water intake monitoring was considered to be a way to lower the risk of bias. The absence of a microbiome analysis was also considered in the other category of risk of bias as it does not offer the possibility to confirm the effects of gut microbiome-targeted interventions directly. The resulting database from applying the modified SYRCLE risk of bias tool was represented as a stacked bar graph (Figure 2). No findings were weighed based on the quality assessment, as the tool does not recommend it.

### 2.4. Synthesis without Meta-Analysis (SWiM)

The Synthesis Without Meta-analysis [76] reporting guideline was used to systematize the results. The recommended complementary checklist can be found in the Supplementary Materials (File S4). Regarding the metrics, Tyrosine hydroxylase (TH) levels in substantia nigra (SN), substantia nigra pars compacta (SNpc), nigrostriatal, striatum, midbrain, or overall brain levels were considered as a metric of dopaminergic cell loss. Since the effect size was not in question due to the high methodological variabilities between studies, the effect direction was considered the primary comparable metric. As a result, even though different studies might have used immunohistochemistry, immunofluorescence, Western Blot, or mRNA expression for the levels of TH+ cells, TH+ fibers, TH protein, or Th mRNA, only the effect direction and the corresponding *p* values were considered for these results. The location where the TH outcome was increased or reduced was also tabulated. The *p* values were uniformized in three significance levels: *p* < 0.05, *p* < 0.01, and *p* < 0.001 to establish three confidence levels for the extracted data. Unless otherwise stated, all results refer to effects produced by the intervention in lesioned animals compared to lesioned controls. A comparable methodology was applied for the ionized calcium-binding adaptor molecule 1 (Iba1), which represents a metric of microglial activation, the glial fibrillary acidic protein (GFAP), which represents a metric of astroglial activation, and the pro-inflammatory IL-6, Interleukin-1beta IL-1β, and Tumor necrosis factor alpha TNF-α. For the motor tests, the metric was the increase or the reduction in performance. If at least one metric for each test was reported as a positive influence, the result was transformed into an increase in performance. In contrast, a negative influence of at least one metric was transformed into a decrease in performance. In the case of multiple statistically significant metrics for each test, the lowest *p*-value was reported. For the microbiome analysis, relative abundance analysis was considered to be relevant, and the presentation of each statistically significant promoted or demoted phyla, order, family, and genera would lead to identifying patterns in the influence of FMT, probiotics, synbiotics, or antibiotics on the relative abundance of gut microbiota. The primary outcomes were tabulated, showing the effect direction for the TH, Iba1, GFAP, IL-1β, IL-6, TNF-α, and the relative abundance of the modified components of the microbiota as well as the significance level as a measure of certainty. A vote count of the effect direction was performed in IBM SPSS, and results were presented with 95% confidence intervals for each outcome. Secondary outcomes were presented in a separate tabulation. In the tabulation format, the articles were arranged based on the type of intervention and the animal disease model used.

## 3. Results

### 3.1. PRISMA Flowchart

A total of 1335 articles were included in the screening process, with 289 being identified as duplicates and eliminated. The number identified per each database can be seen in the PRISMA flowchart (Figure 1). Out of the remaining 1046 articles, 989 were excluded after screening, with two more excluded due to them not being retrieved. Out of the 55 remaining articles, a total of 29 articles were included [41,42,47,77,78,79,80,81,82,83,84,85,86,87,88,89,90,91,92,93,94,95,96,97,98,99,100,101,102] following the eligibility evaluation. One study that met the inclusion criteria due to the high risk of bias was excluded since the number of animals per research group was unclear. The reported results were, in some cases, for more significant numbers of animals, implying incomplete outcome data, reporting biases, and a possible lack of blinding and selection biases [103]. No study using only prebiotics was found to fit the inclusion and exclusion criteria as they were either associated with dietary interventions or the prebiotic status of the interventions was unclear. Out of these 29, 1 article [93] was represented in the tabulation (Table 3) two times as it used two different Parkinson’s disease induction methods.

### 3.2. Quality Assessment

The results of the quality assessment tool can be seen in Figure 2. The results show insufficient information regarding sequence generation, allocation concealment, blinding for performance and detection bias, and random outcome assessment. Performance bias due to housing seems to have the lowest risk overall, with 26 studies reporting data regarding housing conditions such as humidity, temperature, the number of animals per cage, and whether all cages were placed in the same conditions. Regarding the SPF status of the animals, only six studies have used this type of animal, resulting in six low-risk studies for these items. Thirteen studies were considered high risk for other sources because an *ad libitum* administration of the intervention was used or the researchers did not perform a gut microbiome analysis and, as such, could not directly prove the efficiency of the intervention in altering the gut microbiota. The individual quality assessment data for each study can be found in the Supplementary Materials (Table S1).

### 3.3. Studies’ Designs

Regarding the intervention model, out of the 29 articles, 7 studies used an FMT intervention [47,77,78,79,80,81,82], 16 used a probiotic intervention [83,84,85,86,87,88,89,90,91,92,93,94,95,96,97,98], 2 used a synbiotic intervention [99,100] and 4 used an antibiotic intervention [41,42,101,102]. Regarding the donors for the seven FMT studies, two used healthy mice [79,82], two used the sham-operated control [47,77], one used healthy and Parkinson-diseased mice donors [78], one used young mice (YM) and aged mice (AM) as donors [80], and one used human healthy donors as well as PD donors [81]. Out of the sixteen instances with probiotic interventions [83,84,85,86,87,88,89,90,91,92,93,94,95,96,97,98], seven used probiotic mixtures [84,85,87,93,96,97,98], and nine used single strains [83,86,88,89,90,91,92,94,95]. The most common strain was *Lacticaseibacillus rhamnosus* GG (previously *Lactobacillus rhamnosus* GG), used in four studies but only in a mixture with other probiotics (Table 3). One of the mixture studies also investigated each component of the mix separately [87]. For the probiotic interventions that used a single bacteria, *Lactiplantibacillus plantarum* PS128 (previously *Lactobacillus plantarum* PS128) was used in two studies as a single-strain probiotic [86,94]. Two studies used a glucagon-like peptide 1 (GLP-1) producing next-generation probiotic *L. lactis* MG1363-pMG36e-GLP-1 [89,92]. The other strains used individually were *L. plantarum* CCFM405 [95], *L. plantarum CRL 2130* [87], *S. thermophilus* CRL 808 [87], *S. thermophilus* CRL 807 [87], *Agathobaculum butyriciproducens* SR79T [83], *B. breve* CCFM1067 [88], *P. pentosaceus* WMU002 [91], and *Clostridium butyricum* WZMC1016 [83]. The probiotic interventions were predominantly administered via oral gavage, except for three studies that administered them ad libitum in drinking water [92,93,97]. For the two synbiotic interventions, one used *L. rhamnosus* GG combined with polymannuronic acid [100] and the other used *L. salivarius subsp. salicinius* AP-32 in combination with a bacteria-free supernatant obtained from the fermented culture broth [99]. Out of the antibiotic intervention studies, two used antibiotic mixes ad libitum in the drinking water: neomycin, vancomycin, bacitracin, and pimaricin [41], and ampicillin, neomycin, and metronidazole [101], respectively. Of the other two, one used vancomycin [102] while the other used rifamixin [42], both administered via oral gavage. Regarding the timing of the interventions, ten [80,83,84,85,86,92,93,98,101,102] were administered and finished before the induction of the Parkinson’s disease model, nine [42,47,77,79,81,90,91,96,99] interventions were administered after the disease induction was finished, seven interventions [78,82,87,89,94,95,100] were administered simultaneous with the induction model, and three [41,88,97] began before the model induction and continued after the induction.

Regarding the animal models, in 22 instances, C57BL/6 mice were used [47,78,79,80,81,82,83,84,86,87,88,89,90,91,92,93,94,95,97,100,101,102], 2 studies used PD mice [42,96], 2 studies used Sprague Dawley rats [41,99], and 3 used Wistar rats [77,85,98]. When it came to the Parkinson’s disease model, 6-hydroxy dopamine (6-OHDA) was used in six studies, out of which, three had a probiotic intervention [83,84,85], one used FMT [77], one used a synbiotic intervention [99], and one used an antibiotic intervention [41]. 1-methyl-4-phenyl-1,2,3,6-tetrahydropyridine (MPTP) was used in 16 instances, out of which, 8 were with a probiotic intervention [86,87,88,89,90,91,92,93], 5 were with FMT [78,79,80,81,82], 2 with an antibiotic intervention [101,102], and 1 with a symbiotic intervention [100]. Of the 16 studies where MPTP was used, 2 [82,87] used a combined model with probenecid for the induction. Rotenone was also used in four studies, of which three used a probiotic intervention [93,94,95] and one used FMT [47]. The MitoPark mice model was used in two studies, one of which used a probiotic intervention [96] while the other an antibiotic intervention [42]. LPS was used in two probiotic studies [97,98], with one model using a combination with paraquat [97]. Regarding the anesthetic used in the six unilateral stereotaxic 6-OHDAs [41,77,83,84,85,99] and one unilateral stereotaxic LPS model [98], three used the combination of xylazine and ketamine [83,84,98], one used xylazine, tiletamine, and zolazepam [99], one used atropine and pentobarbital [85], one used pentobarbital [77], and one used isoflurane [41].

### 3.4. Primary Outcomes

#### 3.4.1. Dopaminergic Cell Loss

Out of all 29 instances, 5 (17.2%, 95% CI [5.85%, 35.77%]) studies did not report a significant impact [42,83,92,98,102] regarding the dopaminergic TH+ cells, TH protein levels, or mRNA Th levels in the intervention lesioned group compared to the lesioned control. Details regarding the interventions and animal models for these cases can be seen in Table 4. All the other 24 (82.76%, 95% CI [64.23%, 94.15%]) instances found significant differences, with 23 studies [41,47,77,78,79,81,82,84,85,86,87,88,89,90,91,93,94,95,96,97,99,100,101] reporting a positive impact on the amount of TH in the lesioned treatment group compared with the lesioned control and one reporting the positive effects of FMT from AM compared to the FMT from YM [80]. All seven FMT studies reported significant results, with six reporting positive impacts compared to the lesioned control [47,77,78,79,81,82] and one reporting a positive effect of the AM FMT compared to that of the YM FMT [80]. One of the FMT studies also found a negative impact, dependent on the FMT group, namely, the microbiota from Parkinson-diseased human subjects resulted in a worse outcome in both the striatum and the SNpc [81]. In this case, an increased abundance of Akkermansia was also reported. For the probiotic interventions, 13 of the 16 studies reported a positive impact on TH [84,85,86,87,88,89,90,91,93,94,95,96,97], with 3 reporting no significant effect of the intervention in lesioned animals [83,92,98]. Both synbiotic interventions positively impacted all intervention groups compared to lesioned controls [99,100]. Of the four antibiotic intervention studies, two offered an advantage in the survival of dopaminergic cells [41,101], with the other two reporting no statistically significant effect of the intervention in lesioned animals [42,102]. Both antibiotic studies with significant results for this outcome administered the intervention ad libitum in drinking water. When it came to the localization of the positive effect, 21 studies reported reduced dopaminergic cell loss in the SN [47,77,78,79,81,82,84,85,86,87,88,89,90,91,93,94,95,96,97,99,101], with 5 specifically identifying the positive impact in the SNpc [81,85,87,96,97], and 12 [41,78,81,84,85,86,88,93,94,99,100,101] in the striatum as well as 3 less-specific positive findings localized in the midbrain [47,80,100]. Four of the studies that found no significant impact on TH [83,92,98,102] had the intervention administered before the disease model induction and one administered it after [42].

#### 3.4.2. Microglial Activation

Regarding microglial activation, Iba1 was investigated in 16 out of the 29 instances, and 14 (87.5%, 95% CI [61.65%, 98.45%]) [42,47,78,81,82,84,85,86,88,93,94,95,98,102] studies reported a statistically significant reduction in the activation in the intervention lesioned group compared to the lesioned control. Only two (12.5%, 95% CI [1.55%, 38.35%]) reported no statistically significant differences [83,97]. The FMT from PD patients resulted in the only case that reported upregulation in Iba1 expression, including that in the colonic mucosal macrophages [81]. The same study found that an FMT from healthy controls had the opposite effect [81].

#### 3.4.3. Astrocytic Activation

Astrocytic activation was investigated in 13 out of the 29 studies, with 11 (84,62%, 95% CI [54.55%, 98.08%]) [47,78,81,82,83,84,86,88,93,95,102] studies finding a statistically significant reduction in the activation either in the striatal, SN, midbrain, or the entire brain tissue in the intervention lesioned group compared to the lesioned control. Only two (15.38%, 95% CI [1.92%, 45.45%]) studies found no statistically significant differences [80,97]. Out of the studies that reported a reduction in GFAP, one also reported an increase in GFAP in the case of FMTs from PD patients, while transplants from healthy controls had the opposite effect [81].

#### 3.4.4. Cytokines

TNF-α levels were investigated in 16 out of the 29 studies, with 14 (87,5%, 95% CI [61.65%, 98.45%]) [41,42,47,78,79,82,86,87,88,94,95,99,100,102] finding a reduction of this outcome induced by the intervention in lesioned animals and 2 (12,5%, 95% CI [1.55%, 38.35%]) [80,97] finding no statistical significance. Of the studies that report a reduction of TNF-α, 13 reported it in the brain, with 5 of them also finding reductions in the colon (Table 4). One study found it reduced in the serum. IL-1β was investigated in 11 of the 29 studies, with 8 (72.73% 95% CI [39.03%, 93.98%]) [41,42,47,81,82,86,88,95] studies reporting significant findings and 3 [80,97,100] reporting no statistically significant findings. Out of the eight studies, seven reported only reductions in the outcome level. In contrast, one [81] reported both a reduction and increase in levels, depending on the FMT treatment group (Table 4). The FMT from PD patients increased IL-1β expression. At the same time, the FMT from HC resulted in a reduction. IL-6 was investigated in 10 out of 29 studies, with 6 (60% 95% CI [26.24%, 87.84%]) [42,47,86,87,88,95] finding a statistically significant reduction in the brain, serum, or colonic IL-6 levels produced by the intervention in lesioned animals, and 4 (40% 95% CI [12.16%, 73.76%]) [41,80,97,100] reporting no statistical significance.

#### 3.4.5. Behavioral Tests

Out of the 29 studies, 28 investigated the motor effect of the interventions with at least one behavioral test (Table 4). Out of the 28 articles, 27 (96.4% 95% CI [81.65%, 99.91%]) [41,42,47,77,78,79,81,82,83,84,85,86,87,88,89,90,91,92,93,94,95,96,97,98,99,100,102] found a positive impact when comparing the lesioned intervention group to the lesioned control group, and 1 (3.57, 95% CI [0.09%, 18.35%]) [80] found a negative effect of AM FMT compared to YM FMT. Out of the 27, 1 study reported that alongside the positive impact of FMTs from HC, there was also a negative impact of FMTs from PD patients [81].

The Rotarod test was used in 13 studies, with 11 (84,62%, 95% CI [54.55, 98.08]) [47,77,81,82,86,88,91,94,95,96,99] reporting increased performance via the intervention in lesioned animals. One study (7,69%, 95% CI [0.19%, 36.03%]) reported a decrease [80], and one study (7,69%, 95% CI [0.19, 36.03]) [97] reported no significant difference. The Pole descent test was used in 15 studies, with 13 (86,67%, 95% CI [59.54%, 98.34%]) [47,62,78,82,86,87,88,89,90,91,92,95,102] finding a positive impact of the intervention in lesioned animals, 1 (6.67%, 95% CI [0.17%, 31.95%]) [80] reporting a negative impact, and 1 (6.7%, 95% CI [0.17%, 31.95%]) reporting no statistical difference [98]. Traction, grip, or hanging tests were used in five studies, with four (80%, 95% CI [28.36%, 99.49%]) [47,79,89,102] reporting a positive impact of the intervention in the lesioned animals, and one (20%, 95% CI [0.51%, 71.64]%) [80] reporting no significant difference. Apomorphine or an amphetamine-induced rotation test was used in six studies, five (83,33%, 95% CI [35.88%, 99.58%]) [41,77,83,84,99] of which reported a positive impact of the intervention in the lesioned animals, and one (16,67%, 95% CI [0.42%, 64.12%]) reported no significant difference [85]. The open field test was used in eight studies, and all (100%, 95% CI [63.06%, 100%]) [1,2,3,4,5,6,7,8] reported increased performance via the intervention in the lesioned animals. The narrow beam test was used in 11 instances [42,85,86,87,88,90,91,93,94,96,98], all of which reported increased performance via the intervention in lesioned animals (100%, 95% CI [71.51%, 100%]). The cylinder test was used in four studies, with two (50%, 95% CI [6.76%, 93.24%]) [41,93] reporting a positive impact of the intervention in lesioned animals, and two (50%, 95% CI [6.76%, 93.24%]) reporting no significant difference [85,98]. The sticker test was used in two instances; both (100%, 95% CI [15.81%, 100%]) reported a positive impact [47,87]. Gait analysis was used in five studies, with four reporting (80%, 95% CI [28.36%, 99.49%]) a positive impact [41,42,93,96], and one (20%, 95% CI [0.51%, 71.64%]) study with no statistical difference [85].

#### 3.4.6. Microbiome Relative Abundance

In 19 out of the 29 studies, the relative abundance of gut microbiota was investigated, with 17 reporting (89.47%, 95% CI [66.86%, 98.70%]) significant differences in the species’, order’s, family’s, or genera’s relative abundances [42,47,62,78,80,82,86,88,90,91,94,95,97,99,100,101,102]. Two studies (10.53%, 95% CI [1.3%, 33.14%]) reported no significant differences [41,92].

Bacteria from the *Bacillota* (*Firmicutes*) phylum and its subclassifications are reported in 15 studies, with 1 study [80] reporting significant differences between treatment groups, with FMTs from AM increasing the abundance of Lactobacillus and Duboisella as well as a decrease in *Ruminococcacea* UCG-014 compared to FMTs from YM. The other 14 studies report significant differences in the intervention lesioned mice compared to the lesioned controls, with four of the studies reporting only increased abundances as follows: *Lactobacillaceae*, *Butyricicoccus*, and *Roseburia* [47], *Lactobacillus plantarum* [86], *Ruminiclostridium 6* and *Acetatifactor* [94], and *Streptococcaceae* [97]. Two reported only reduced abundances: the *Eubacterium xylanophilum* group and *Lachnospiraceae* unclassified [81], and *Dubosiella* and *Lactobacillus* [88]. The other eight reported components of the phylum being influenced in both directions. When considering only the phylum level, three studies reported an overall decrease in abundance, but in each of these cases, other components of the phylum were increased: *Faecalibaculum* and *Turicibacter* [95], *Lachnospiraceae* [91], and *Robinsoniella* and *Dorea* [101]. For one of these studies [91], the decrease in the overall abundance of the phylum also coincided with the reduction of *Dubosiella* and *Enterococcus*. Sun et al. [78] reported an increase in the *Eubacteriales* (*Clostridiales*) order but a decrease in *Erysipelotrichales* (*Turicibacteriales*). Liu et al. [100] reported that for the synbiotic group, there was an increase in *Lactobacillales* and a reduction in *Erysipelotrichales (Turicibacteriales).* Zhang et al. [82] reported an increase for *Blautia* and a decrease for *Anaerostipes*, *ASF356*, and *Ruminococcus*. Tsao et al. [99] reported increased *Ruminococcaceae* and reduced *Balutia, Coprococcus*, and *Eubacterium* for the synbiotic and probiotic groups. Cui et al. [102] reported an increase in *Blautia* and *Ileibacterium* and a reduction in *Duboisiella*.

Bacteria from the *Bacteriodota* (*Bacteroidetes*) phylum and its subclassifications are reported in nine studies, with one study [80] reporting significant differences between treatment groups, with FMTs from AM increasing the abundance of *Odoribacter* compared to FMTs from YM. The other eight found significant alterations produced by the intervention in lesioned mice, with three reporting increased abundance and the rest reporting lowered abundance. Of the three, two report increases at the phylum level and increases in *Muribaculaceae* [91] and *Parabacteroides* [101]. The other study of the three reported an increase in the *Barnesiella* genus [47]. The lowered abundances induced by the interventions in lesioned animals are as follows: *Bacteroides* [88,100], *Prevotellaceae_NK3B31*, *Alistipes*, and *Odoribacter* [90], *Bacterioidetes* phylum, specifically *Alistipes* [95], and *Prevotellaceae UCG-001* [42].

Bacteria from the *Actinomycetota* phylum and its subclassifications are reported in eight studies, with one study [80] reporting significant differences between treatment groups, with FMTs from AM increasing the abundance of *Parvibacter* compared to FMTs from YM. Out of the other seven studies, three found increased abundance in the treated lesioned group compared to the lesioned control, three found reduced abundance, and one saw both increases and reductions in different components of the phylum. Three studies report increased abundances for *Bifidobacterium* [88,94,95], with one also finding increases for *Adlercreutzia* [94]. The reduced abundances are as follows: *Bifidobacterium* [82], *Propionibacterium* for the probiotic group [99], *Coriobacteriales*, and *Bifidobacteriales*, namely *Bifidobacterium* [102].

Six studies report bacteria from the *Pseudomonadota* (Proteobacteria) phylum and its subclassifications. One study [80] reported significant differences between treatment groups, with FMTs from AM increasing the abundance of Proteobacteria compared to FMTs from young mice. All the others found decreased abundances of *Proteobacteria* [78,82,91], *Enterobacteriaceae* [86], and *Escherichia-Shigella* [88].

For the *Verrucomicrobiota* phylum, only the *Akkermansia* genus was found to have altered in the treated lesioned group compared to the lesioned control. Of the six studies that reported significant differences, four reported increases in *Akkermansia* relative abundance [81,88,90,102] and two reported reduced abundance [47,95]. In one study, the increase in abundance was found in the group that received FMTs from PD human patients [81].

Bacteria from the *Thermodesulfobacteriota* (*Desulfobacterota*) phylum and its subclassifications are reported in four studies. Of the other four studies, one found decreases in *Bilophila* [95], and two found reduced abundance for *Desulfovibrio* [47,81]. In contrast, one study [80] reported significant differences between treatment groups, with FMTs from AM increasing the abundance of *Desulfovibrionales*. One of the reduced abundances for *Desulfovibrio* was reported in mice that received FMTs from healthy human controls [81].

Two studies report bacteria from the Campylobacteriodota phylum and its subclassifications. One study [80] reported significant differences between treatment groups, with FMTs from AM increasing the abundance of Helicobacter and Campylobacter compared to FMTs from YM. The other [47] found an increased abundance for Helicobacter when comparing the lesioned treatment group to the lesioned control.

Bacteria from the *Mycoplasmatota* (*Tenericutes*) phylum are reported to have significant differences between the two studies. One study [80] reported significant differences between treatment groups, with FMTs from AM increasing the abundance of *Anaeroplasma* compared to FMTs from YM. The other study [82] found reduced amount of *Tenericutes* phylum when comparing the lesioned treatment group to the lesioned control.

### 3.5. Secondary Outcomes

The secondary outcomes are tabulated in Table 5. Four studies reported a reduction in the expression or aggregation of a-syn [47,79,89,91] in the intervention lesioned cohort compared to the lesioned control. In five instances, ZO-1, occludin, and/or claudin tight junction proteins were reported with statistical significance, with two studies showing upregulation [47,95] and three showing downregulation [42,88,89] in the lesioned intervention group compared to the lesioned control. BDNF increases in the lesioned intervention group compared to the lesioned control were reported in seven instances [84,86,89,93,100,104]. GDNF increases in the lesioned intervention group compared to the lesioned control were reported in seven cases [84,86,89,93,100,104]. TLR4 was reported to have decreased in five studies [47,79,90,92,102]. The anti-inflammatory IL-10 was increased in four studies [82,87,88,94]. NF- κB was reported with decreases in five studies [47,79,84,90,102]. Three studies reported lowered iNOS levels [48,83,95], while COX2 was reported lowered in two studies [42,48].

Phosphorylated Akt or the phosphorylated Akt to non-phosphorylated Akt reported was found to be increased in three studies [83,84,93] and decreased in one study [79]. Phosphorylated Pi3K to nonphosphorylated reported was found to be decreased in one study [79] and increased in one study [93], also with nonphosphorylated Pi3K being reported as increased in another study [84].

Regarding the SCFA profile, five studies [79,88,90,99,100] reported significant changes in the lesioned intervention group compared to the lesioned control group. One study [80] reported differences between AM FMT and YM FMT, with AM FMT lowering acetic acid (AA), butyric acid (BA), isobutyric acid (IBA), propionic acid (PA), and valeric acid (VA). Out of the five studies, two reported reduced levels; Zhong et al. [79] reported lowered AA, PA, BA, and N-valeric acid (N-VA), and Sun et al. [90] reported reduced AA, PA, BA, and VA. The other three reported increases in levels as follows: Li et al. [88] reported increased levels of AA, BA, IBA, and isovaleric acid (IVA), Tsao et al. [99] reported an increase in levels of total SCFA, and PA, BA in the symbiotic, probiotic and prebiotic groups, Liu et al. [100] reported an increase in the probiotic group for AA and BA.

## 4. Discussion

### 4.1. Metodological Considerations

The findings of our review must be discussed in the context of the natural evolution of PD and the disease models that aim to replicate it. There are multiple pathogenetic mechanisms intricated in PD development. Firstly, α-syn proteostasis is disturbed by deficits of the neuronal autophagy-lysosomal pathway [105], causing α-syn secretion and its subsequent uptake in adjacent neurons to form Lewy-like aggregates [5]. Secondly, mutations in the PTEN-induced kinase 1 (PINK1) and parkin RBR E3 ubiquitin-protein ligase (PRKN) genes disturb mitochondrial autophagy, which facilitates dopaminergic neuron loss [106,107]. Damaged neurons also release mitochondrial molecules, which act as damage-associated molecular patterns (DAMPs) and initiate gliosis [108,109]. Thirdly, immunosenescence-related chronic neuroinflammation itself may promote neuronal death in the SNpc and striatum through microglial tumor necrosis factor (TNF), interleukin 1β (IL-1β), transforming growth factor (TGF) β, IL-6, reactive oxygen (ROS) and nitric oxide species [110].

Rodent PD induction methods can be toxic, inflammatory, or genetic, and aim towards replicating the mitochondrial dysfunction directly by affecting mitochondrial autophagy or indirectly through inflammation. The reviewed studies most commonly used the neurotoxic agent MPTP, which leads to dopaminergic lesions by blocking the complex I of the respiratory chain. This agent is administered systemically and induces a bilateral model with the disadvantage that it does not replicate α-syn aggregation and is challenging to use in rats [111]. Another model is related to 6-OHDA administration, a compound administered stereotaxically, unilaterally or bilaterally, which disrupts complex I and induces supplementary oxidative stress without α-syn dysfunction [111]. Rotenone is another agent that inhibits complex I and triggers α-syn aggregation without dopaminergic solid neurodegeneration and motor deficit development [111]. The LPS model is an inflammatory model that also results in α-syn aggregation [112]. An increasingly popular genetic model is the MitoPark mouse, wherein the mitochondrial transcription factor A is knocked out in the midbrain dopaminergic neurons [113]. Our findings show that despite the differences between these methods, gut-targeted interventions can mitigate the damaging effects of the models at multiple levels by altering the gut microbiota, with some caveats. Regarding the efficiency of the interventions, all FMT studies resulted in statistically significant results; all of them show a reduced dopaminergic loss, with one study also reporting an increase for the FMT from PD patients. Despite the two different induction models, both symbiotic studies found lowered dopaminergic cell loss for the probiotic, prebiotic, and symbiotic groups. Of the probiotic interventions, three studies [83,92,98] did not find significant differences induced by dopaminergic cell loss, but they found increased motor performance. Fang et al. [92] used an MPTP model and found no significant changes in relative abundance. This lack of result might be linked to the methodology since they used an *ad libitum* administration of the probiotic in the drinking water. For the antibiotic studies, two authors [42,102] found no positive effect on the dopaminergic cell loss produced by the MitoPark model and the MPTP model despite finding increased motor function and significant changes in relative abundance. The fact that there are studies showing increased motor outcomes despite not having better dopaminergic outcomes could be because gut-targeted interventions might have an impact on the motor outcomes downstream from the dopaminergic cell death. This seems plausible since almost all the studies where the intervention had no impact on dopaminergic outcomes also lowered either astrocytic activation or lowered cytokines. This could mean that the interventions have a level of anti-inflammatory effect that could facilitate better motor function, but is insufficient to prevent dopaminergic cell death.

### 4.2. Main Findings

The study showed that most reviewed papers report a reduction via gut-targeted interventions in the adverse effects induced by the disease on the reviewed primary outcomes. Reduced dopaminergic cell loss (82.76%, 95% CI [64.23%, 94.15%]), reduced microglial activation (87.5%, 95% CI [61.65%, 98.45%]), reduced astrocytic activation (84,62%, 95% CI [54.55%, 98.08%]), and reduced pro-inflammatory cytokines as well as increased performance in motor tests (96,4% 95% CI [81.65, 99.91]) induced by the gut-targeted interventions were noticed for a large majority of the studies.

When discussing how these results correlate with the alteration of the gut microbiota, it is essential to mention that PD presents two subtypes: body-first PD, where the α-synuclein pathology is transmitted in a bottom-up fashion from the gut to the central nervous system (CNS), causing prodromal constipation and rapid eye movement sleep behavior disorder, and brain-first PD, which lacks these clinical features [114]. Besides the possible origination of the disease in the gut in body-first PD, the microbiome mediates PD-related neuroinflammation through multiple pathways. The first uses bacterial component-sensitive Toll-like receptors (TLRs) in neurons and CNS immune cells [50,115]. TLR2, TLR9, and TLR4, which binds LPS explicitly, are the most important in PD; downstream, they use myeloid differentiation primary response protein 88 (MyD88) or the TIR-domain-containing adaptor-inducing interferon-β (TRIF) pathway to initiate proinflammatory cytokines IL-1, IL-6, and TNF-α synthesis [116]. Regarding this pathway, our review included five studies that reported low TLR4 expression, outcomes associated with lowered dopaminergic cell loss in three of the studies, and better motor outcomes in three. One study reported the reduction of TLR4 despite not having had an impact on the two primary outcomes. IL-6 decreased in five out of nine studies, in the brain in four cases, three in the colon, and one in the serum. TNF-α was investigated in 15 studies, with 13 finding a reduction in this outcome produced by the intervention. The reduction was localized to the brain in 12 cases, the colon in 5 cases, and serum in 1 case.

The second pathway is related to the proinflammatory [117] nuclear factor-kappa B (NF-κB) signaling pathway, whose activation by misfolded α-syn promotes apoptosis in neurons and induces pro-inflammatory cytokine release in glial cells [118]. Canonical NF-κB activation uses the IκB kinase complex, and the main factors involved are cytokines like TNF- α and bacterial LPS. In turn, the NF-κB released can stimulate TNF-α and IL-1β production [119,120]. Another possible pathway is the one that uses the microglial nucleotide-binding oligomerization domain-like receptor (NLR) family pyrin domain containing 3 (NLRP3) inflammasome, which includes a sensor, a recruitment domain, and the caspase-1 protease [121]. An initial proinflammatory stimulus like the fibrillary α-synuclein-TLR2 interaction or LPS-TLR4 binding increases NLRP3 transcription and pro-IL-1β and pro-IL-18 secretion. Subsequently, a secondary pathogen-associated molecular pattern (PAMP) or DAMP signal triggers NLRP3 assembly and IL-1β/IL-18 secretion [122,123,124]. Our review found five studies where NF-κB was reduced regarding this pathway. This result was associated with lowered dopaminergic cell loss and better motor outcomes in three of the five studies. One study did report the reduction in NF-κB despite no impact on the two main results. IL-1β was reduced by the intervention in eight out of the eleven studies, with six finding the reduction in the brain, four in the colon, and one in the serum.

By contrast, the PI3K/AKT pathway plays a vital role in neuroprotection [125]. Phosphatidylinositol 3-kinases (PI3Ks) operate downstream from receptor tyrosine kinases and G protein-coupled receptors and ensure the intracellular propagation of cytokine and growth factor signals by activating the serine/threonine kinase AKT pathway [126]. Downstream, PI3K-AKT inhibits IL-β, IL-6, and (interferon-gamma (IFN-γ) production and increases the levels of anti-inflammatory cytokines like IL-10 [127]. This pathway suppresses neuronal apoptosis, and PD patients have significantly decreased phosphorylated AKT levels in the SNpc [128]. Our findings report a reduction in phosphorylated Akt in three of the four studies, and phosphorylated PI3K/PI3k decreased in one of the two studies. IL-10 was increased in four of the studies that investigated it.

In conclusion, the gut microbiome-targeted interventions altered all the previously described pathways. How specific relative abundances might influence these results is less clear. The relationship between gut dysbiosis and PD pathogenesis is mediated through several mechanisms. Firstly, high LPS levels produced by Gram-negative bacteria impact the ENS, causing hypophagia and weight loss, inflammation via Toll-like receptors, gut ⍺-syn pathology, and eventually, BBB disruption [15,21,23]. Secondly, the microflora secrete microbial amyloid protein, which promotes ENS α-syn aggregation and inflammation, and hippuric acid, which can cross the BBB and negatively influence disease status [22]. Thirdly, a fraction of bacterial SCFAs enter the bloodstream and help regulate BBB integrity by restoring junctional proteins and neuroinflammation [129]. Low SCFA-producing bacteria levels are correlated with PD constipation, with its severity being a predictor of cognitive and motor decline [22]. Lastly, the microbiota impacts host neurotransmitter production directly through bacterially generated γ-aminobutyric acid and indirectly by influencing tryptophan metabolism [13]. Significantly, bacteria like *Enterococcus faecalis, Eggerthella lenta*, and *Clostridium sporogenes* interfere with levodopa metabolism and absorption, lowering its efficacy [130,131].

Two bacterial genera are commonly related to gut homeostasis: *Akkermansia* and *Bacteroides. Akkermansia* levels, in particular, are strongly correlated with gut dysbiosis [132]. *Akkermansia muciniphila* is a mucin-degrading species that promotes gut barrier integrity by stimulating mucus production and using its metabolic byproducts to cross-feed other microbiota species [133]. *A. muciniphila*-derived extracellular lipid vesicles support tight junction integrity by activating the AMP-activated protein kinase, which contributes to tight junction assembly and increasing occludin and claudin four expression [134,135,136]. Lastly, it exerts pro- and anti-inflammatory properties through an outer membrane pili-like protein named MucT, which induces IL-8, IL-6, IL-1β, IL-10, and TNF-α production [137]. Overall, increases and decreases in A. muciniphila abundance ultimately disturb mucus layer thickness and intestinal barrier integrity [34,138]. The ambiguous relation between *Akkermansia* relative abundance and inflammation is also seen in our results, with two studies reporting decreased relative abundances, and four studies reporting increased abundances. Still, one of the four studies where increased abundance was observed was in the PD FMT group, which also saw increased inflammation and dopaminergic cell loss and lowered motor outcomes.

The second genus, *Bacteroides* spp., is also significant to host health and disease [139]. Firstly, this genus exerts immunomodulatory effects through capsular polysaccharide A (PSA), which supports CD4+ T-cell development, T-helper cell regulation, and IL-10 production [140]. Secondly, the commensal *Bacteroides fragilis* contributes to gut homeostasis by enhancing the relative abundance of *A. muciniphila* and, thereby, gut barrier integrity [141]. Further, *B. fragilis* helps prevent chronic inflammation and produces short-chain fatty acids (SCFAs), negatively regulating the NLRP3 inflammatory pathway [142]. Our results regarding the *Bacteroides* seem to contradict these results, as two studies reported the lowered *Bacteroides* genus being associated with better outcomes. This could be because not all species of the genus are thought to be beneficial, with some possibly promoting inflammation [143] or possible differences in animal responses.

Regarding another genus that is often associated with gut health through the upregulation of anti-inflammatory cytokines [144], three of the reviewed studies found increases in the relative richness of Bifidobacterium, while two found a reduction.

The results for the *Pseumonodota* phylum are the clearest. Five studies reported decreased relative abundance, with only one study finding an increase—in the AM FMT group, it was associated with worse motor consequences.

### 4.3. Future Research

The reviewed studies found a reduction in the expression or aggregation of a-syn [47,79,89,91]. The tight junction proteins were reported as upregulated in two studies [47,95] and in three studies, downregulated [42,88,89], suggesting the possible involvement of the tight junction in preventing PD advancement through microbiome alteration. This is further sustained because seven of the seven studies investigating BDNF found it to be increased [84,86,89,93,100,104]. Previous studies showed that BDNF plays a role in the modulation of tight junction proteins and the regulating the intestinal barrier. Another avenue of future research could consist of looking towards early signs of PD development, such as sleep disorders, and how these might play a role in gut dysbiosis in PD [145]. Given the increasing worldwide prevalence of sleep disorders [146], it is necessary to address how much this risk factor might contribute to the pathogenesis of PD. Future studies should further explore the relation between different bacterial genera, microbial metabolites, and the intestinal tight junctions to better contextualize positive clinical results and provide clear molecular pathways that might influence PD pathogenesis. Also, our findings show solid prospects for gut microbiome-targeted interventions as disease-modifying interventions in animal PD models. Future research should clarify if these findings could be replicated in human patients.

### 4.4. Limitations

The main limitation of our study was uncovered by our quality analysis, showing methodological inconsistencies for many of the studies included in the review. The fact that most studies did not use SPF animals did not confirm the microbiome alteration of the intervention and have not clearly stated how the blinding or the allocation concealment was performed raises the possibility of the non-replicability of these findings. The non-replicability of at least part of the findings would alter the results of our systematic review. Further, the limitation of the synthesis must be taken into account. The data transformation aims to make data more presentable and comparable but might result in losing key details that might lead to further insights. Limiting the synthesis to narrative methods, without metanalysis, could not offer a weighted, quantitative result and could not inform us of the effect size. Nonetheless, using p levels does offer us some measure of the certainty of the findings.

## 5. Conclusions

This systematic review shows that non-dietary gut microbiome-targeted interventions consistently improve neuroinflammatory and motor outcomes in PD animal models and should be considered candidates for future disease-modifying interventions in human studies.

## Figures and Tables

**Table 1:** Inclusion and exclusion criteria.

Inclusion Criteria	Exclusion Criteria
In vivo murine Parkinson’s disease models	In vitro, in silico, in vivo human or non-murine models, all review articles
Intervention—Fecal microbiota transplantation, Antibiotic, Prebiotic, Probiotic, or Synbiotic supplementation	Dietary, nutraceutical, fatty acids, and fatty acid derivate interventions.
Outcomes: dopaminergic cell loss; at least one of the others: cytokines, neuroinflammatory markers, or motor behavior tests	High risk of bias

**Table 2:** Summary of the data extraction template.

Data Category	Outcomes	Extracted data
Study design	Parkinson’s induction model	Animals and induction substance
		Dose, time, frequency, and route of administration
		Other data (vehicle, anesthetics, attrition)
	Intervention	Type of intervention—Probiotic strain, FMT donor, Antibiotics
		Dose, time, frequency, and route of administration
		Other data (vehicle, anesthetics, attrition)
Primary outcomes	Dopaminergic cell loss	Tyrosine Hydroxilase (TH)
	Microglial activation	Ionized calcium-binding adaptor molecule 1 (Iba1)
	Astrocyte activation	Glial fibrillary acidic protein (GFAP)
	Cytokines	IL-6, IL-1β, TNF-α
	Motor behavioral tests	Rotarod test, Narrow beam test, Pole test, Open field test
		Traction test, Grip strength, Hanging test, Cylinder test
		Apomorphine-induced/Amphetamine-induced rotation test
		Gait analysis, Elevated body swing test, Sticker test
	Microbiome analysis	Relative abundance analysis
Secondary outcomes	Cytokines	IL-10, IL-1α
	Neuroinflammation markers and signaling pathways	BDNF, Glial cell line-derived neurotrophic factor (GDNF), Proliferator-activated receptor γ (PPaR γ), Cyclooxygenase-2 (COX2), iNOS, PI3K, Protein kinase B (Akt), NF-κB
	Microbiome analysis	Alpha and beta diversity
	Other outcomes	α-syn, LPS, ZO-1, Occludin, Claudin, SCAF

**Table 3:** Probiotic mixtures used as interventions.

Study	Probiotic Strains
Castelli et al. [84]	*S. thermophilus*, *B. longum*, *B. breve*, *B. infantis*, *L. acidophilus*, *L. plantarum*, *L. paracasei*, *L. Delbrueckii subsp. bulgaricus*, *and L. brevis*
Cuevas-Carbonell et al. [85]	*L. rhamnosus* GG, *B. animalis subsp. lactis* BB-12
Dwyer et al. [97]	*L. plantarum*, *L. delbrueckii subsp. bulgaricus*, *L. paracasei*, *L. acidophilus*, *B. breve*, *B. longum*, *B. infantis*, *and S. salivarius subsp. thermophilus*
Hsieh et al. [96]	*B. bifidum*, *B. longum*, *L. rhamnosis*, *L. rhamnosus* GG, *L. plantarum* LP28, *and L. lactis subsp. lactis*
Parra et al. [98]	*L. rhamnosus* GG, *B. animalis subsp. lactis* BB-12
Perez Visnuk el al [87]	*L. plantarum* CRL 2130, *S. thermophilus* CRL 808, *and S. thermophilus* CRL 807
Srivastav et al. [93]	*L. rhamnosus* GG, *B. animalis subsp. lactis* BB-12, *and L. acidophilus* LA-5

**Table 4:** Tabulation of primary outcomes for each study included in the systematic review.

Study, Year	PD Model	Interv.	TH	Iba-1	GFAP	IL-6	TNF-alfa	IL-1beta	Behavioural Test Performance	Gut Microbiome Relative Abundance
Yu et al., 2023 [77]	6-OHDA, rat	FMT	SN: ↑ *						RR: ↑ ***; APO: ↑ ***; OF: ↑ **	
Sun et al., 2018 [78]	MPTP, mice	FMT	SN ↑ **; STR ↑ *	SN ↓ ***	SN ↓ ***		STR ↓ **; Colon ↓ **		PT: ↑ ***	↑ Clostridiales * ↓ Proteobacteria **; Turicibacterales *, Enterobacterales **
Zhong et al., 2021 [79]	MPTP, mice	FMT	SN IHC ↑ **; WB ↑ ***				SN ↓ **; STR ↓ *		TT: ↑ ***; PT: ↑ **	
Qiao et al., 2023 [80]	MPTP, mice	FMT	AM + MPTP IF ↑ ^##^, WB ns.		ns	SN ns; Colon ns	SN ns; Colon ns	SN ns; colon ns	RR: AM + MPTP ↓ ^#^, TT ns; PT: AM + MPTP ↓ ^##^	*AM—*↑ *Lactobacillus* ^##^; ↑ *Proteobacteria* ^#^, ↑ *Desulfovibrionales* ^#^ ↑ *Dubosiella* ^#^, ↑ *Helicobacter* ^#^, ↑ *Campylobacter* ^#^, ↑ *Odoribacter* ^#^, ↑ *Parvibacter ^#^, * ↑ *Anaeroplasma* ^#^ ↓ *AM—Ruminococcacea* UCG-014 ^##^, ↓ *Eubacterium xylanophilum* group ^#^
Xie et al., 2023 [81]	MPTP, mice	FMT	STR PD ↓ *, HC ↑ ***; SNpc PD↓ *** HC↑ ***	SNpc PD ↑ ***, HC ↓ **; STR PD ↑ *** HC ↓ **; Colon PD ↑ ***, HC ↓ **	SNpc PD ↑ *** HC ↓ ***; STR PD ↑ ***; HC ↓ ***			Colon: PD ↑ **, HC ↓ *	RR: PD ↓ **; HC ↑ ***; PT: PD ↓ ***; HC ↑ ***.	PD: ↑ *Verrucomicrobiota* *** ↑ *Akkermansia* *** ↓ *Eubacterium xylanophilum group* **, ↓ *Lachnospiraceae unclassified* * HC: ↓ *Desulfobacterota* **, *Desulfovibrio* **
Zhang et al., 2021 [82]	MPTP + probenecid, mice	FMT	SN ↑ *	SN M1 ↓ ***			SN ↓ **	SN ↓ *, Colon: ↓ *	RR: ↑ *; PT: ↑ *; OF: ↑ **	↑ Blautia * ↓ *Proteobacteria* **, ↓ *Tenericutes* *; ↓ *Anaerostipes* **, ↓ *Bifidobacterium* *, ↓ ASF356 **, ↓ *Ruminococcus* **
Zhao et al., 2021 [47]	Rotenone, mice	FMT	SN: IF ↑ ***, Mid-brain: WB ↑ *	SN ↓ ***	SN ↓ ***	Mid-brain ↓ ***; SN ↓ *; Colon: ↓ ***	Mid-brain: ↓ ***; SN ↓ **; Colon: ↓ ***	Mid-brain: ↓ ***; SN ↓ *; Colon: ↓ ***	RR: ↑ ***; GT: ↑ **; PT: ↑ **; ST: ↑ ***.	↑ *Proteobacteria* **, ↑ *Helicobacteraceae* ***, ↑ *Enterobacteriaceae* *, ↑ *Lactobacillaceae* **; ↑ *Barnesiella* *, ↑ *Butyricicoccus* **, ↑ *Helicobacter* ***, ↑ *Roseburia* ** ↓ *Verrucomicrobia* *** ↓ *Akkermansia* ***, ↓ *Coriobacteriaceae* **, ↓ *Desulfovibrio* *
Lee et al., 2022 [83]	6-OHDA, mice	PRO	ns	ns	SN ↓ **				AMPH: ↑ *	
Castelli et al., 2020 [84]	6-OHDA, mice	PRO	STR ↑ *; SN ↑ **	STR ↓ **	STR ↓ *				APO: ↑ **; EBST: ↑ ***	
Cuevas-Carbonell et al., 2022 [85]	6-OHDA, rats	PRO	STR ↑ ***; SNpc ↑ *	STR ↓ **					BT: ↑ *; APO: ns; CT: ns; GA: ns.	
Liao et al., 2020 [86]	MPTP, mice	PRO	SN ↑ ***; STR ↑ ***	STR: ↓ **	STR ↓ *	STR ↓ **	STR ↓ ***	STR ↓ **	RR: ↑ ***; PT: ↑ ***; BT: ↑ ***	↑ *Lactobacillus plantarum* *** ↓ *Enterobacteriaceae* ***
Perez Visnuk et al., 2020 [87]	MPTP + probenecid, mice	PRO	SNpc Mix ↑ *			Serum Mix ↓ *	Serum Mix ns; Brain: Mix ↓ *		PT: Mix ↑ *, 2130 ↑ *, 807 ↑ *, 808 ↑ *; BT: Mix ↑ *, 2130 ↑ *, 807 ↑ *, 808 ↑ *; ST: Mix ↑ *	
Li et al., 2022 [88]	MPTP, mice	PRO	SN ↑ ***; STR ↑ ***	STR ↓ ***	STR ↓ ***	STR ↓ ***; Colon ↓ ***	STR ↓ ***; Colon ↓ ***	STR ↓ ***; Colon ↓ *	RR: ↑ **; PT: ↑ ***; OF: ↑ ***; BT: ↑ ***	↑ *Akkermansia* *, ↑ *Bifidobacterium* ** ↓ *Bacteroides* **, ↓ *Escherichia-Shigella* *, ↓ *Dubosiella* **, ↓ *Lactobacillus* **
Yue et al., 2022 [89]	MPTP, mice	PRO	SN IHC↑ *; WB ↑ **						HT: ↑ ** PT: ↑ **; OF: GLP-1 ↑ **	
Sun et al., 2021 [90]	MPTP, mice	PRO	SN ↑ *						PT: ↑ **; OF: ↑ **; BT: ↑ **	↑ *Verrucomicrobia* **; ↑ *Akkermansia:* *, ↓ *PrevotellaceaeNK3B31* **; ↓ *Alistipes* *; ↓ *Odoribacter* *
Pan et al., 2022 [91]	MPTP, mice	PRO	SN ↑ **						RR: ↑ **; PT: ↑ **; BT: ↑ **	↑ *Bacteroidota* **, ↑ *Muribaculaceae* **, ↑ *Lachnospiraceae* *, ↑ *Defluviitaleaceae* *, ↓ Proteobacteria **, ↓ Firmicutes *, ↓ Erysipelotrichaceae *, ↓ Enterococcaceae **, ↓ Dubosiella *, ↓ Enterococcus **
Fang et al., 2020 [92]	MPTP, mice	PRO	ns						PT: ↑ *; OF: ↑ **	ns
Srivastav et al., 2019 [93]	MPTP, mice	PRO	SN ↑ *, STR ↑ **	SNpc ↓ **	SNpc ↓ **				BT: ↑ *; CBT ↑ ***; GA: ↑ *; CT: ↑ ***	
Srivastav et al., 2019 [93]	rotenone, mice	PRO	SN ↑ **, STR ↑ **	SNpc ↓ ***	SNpc ↓ ***				BT: ↑ *; CBT ↑ ***; GA: ↑ ***; CT: ↑ ***	
Lee et al., 2023 [94]	rotenone, mice	PRO	SN ↑ **; STR ↑ **	SN ↓ *			SN ↓ ***		RR: ↑ *; Beam: ↑ ***	↑ *Bifidobacterium* ***, ↑ *Ruminiclostridium 6* ***, ↑ *Adlercreutzia* ** ↑ ASF356 ** ↑ *Acetatifactor* *
Chu et al., 2023 [95]	rotenone, mice	PRO	SN ↑ *	Nigro-striatal ↓ **	Nigro-striatal ↓ *	Mid-brain: ↓ **; Colon ↓ *	Mid-brain ↓ **; Colon: ↓ **	Mid-brain ↓ *; Colon: ns	RR: ↑ *; PT: ↑ 50 cm **, 70 cm *; OF: ↑ 5 mm *; ↑ 10 mm **	↑ *Actinobacteria* ***, ↑ Bifidobacterium *, ↑ *Faecalibaculum* *, ↑ *Turicibacter* * ↓ *Firmicutes* *, ↓ *Bacteroidetes* ***, ↓ *Alistipes* *, ↓ *Akkermansia* *, ↓ *Bilophila* *, ↓ *Ruminococcaceae* UCG 004 *, ↓ *Ruminococcaceae* UCG 009 *
Hsieh et al., 2020 [96]	MitoPark PD, mice	PRO	SNpc ↑ *						RR: ↑ ***; BT: ↑ ***; GA: ↑ *	
Dwyer et al., 2021 [97]	LPS + PQ, mice	PRO	SNpc ↑ **	ns	ns	ns	ns	ns	RR: -; Micromax: -	↑ *Streptococcaceae* ***
Parra et al., 2023 [98]	LPS, rats	PRO	ns	SN ns; STR ↓ *;					PT: NS; BT: swing phase ↑ **; CT: ns	
Tsao et al., 2021 [99]	6-OHDA, rats	SYM	STR: PRO ↑ *, PRE ↑ * SYN ↑ *; SN: PRO ↑ *, PRE ↑ * SYN ↑ *;				STR PRO ↓ *, PRE ↓ * and SYN ↓ *		RR: ↑ RO *, PRE *, SYM *; APO: ↑ PRO *, PRE * and SYM *	SYN: ↑ Ruminococcaceae *, ↓ *Aggregatibacter* *, ↓ *Balutia* *, ↓ *Coprococcus* *, ↓ *Eubacterium* *, ↓ *Prevotella* * PRO: ↑ *Ruminococcaceae* *, ↓ *Propionibacterium* *, ↓ *Clostridium* * PRE: ↑ *Prevotella* *, ↑ *Elizabethkingia* *, ↑ *Eggerthella* *, ↑ *Faecalibacterium* *, ↑ *Mitsuokella* *, ↑ *Succinatimonas* *, ↑ *Bifidobacterium* *, ↓ *Lactobacillus* *
Liu et al., 2022 [100]	MPTP, mice	SYM	Midbrain: SYN ↑ **; STR: SYN ↑ ***, PRE ↑ *, PRO ↑ *			STR ns	STR PRE ↓ ***	STR ns	GT: SYN ↑ **; OF: PRE *, PRO: * ↑, SYN: ↑ ***;	SYM: ↑ *Lactobacillales* *, ↑ *Lactobacillus* *, ↓ *Bacteriodaceae* **, ↓ *Bacteroides* ** PRO: ↑ *Clostridia*, ↑ *Ruminococcaceae* **, ↓ *Lactobabilalles* *, ↓ *Lactobacillus* * PRE: ↓ *Lactobabilalles* **, ↓ *Lactobacillus* ** all groups ↓ *Turicibacterales, * ↓ *Turicibacter* ***
Koutzoumis et al., 2019 [41]	6-OHDA, rat	ATB	STR ↑ **; SN ns			STR ns;	STR: ↓ *	STR: ↓ *	AMPH: ↑ *; GA: ↑ *; CT: ↑ *	ns
Hong et al., 2022 [42]	MitoPark PD, mice	ATB	ns	SN ↓ *		Serum ↓ *	Serum ↓ *	Serum ↓ *	BT: ↑ **; GA: ↑ *	↓ *Prevotellaceae* UCG-001 **
Pu et al., 2019 [101]	MPTP, mice	ATB	SN, STR ↑ **							↑ *Bacteroidetes* ***, ↑ *Proteobacteria* ***; ↑ *Robinsoniella* *, ↑ *Dorea* *, ↑ *Parabacteroides* *** ↓ *Firmicutes* **; ↓ *Lactobacillus* ***
Cui et al., 2023 [102]	MPTP, mice	ATB	ns	SNpc ↓ ***	SNpc ↓ ***		STR ↓ ***; Colon↓ *		TT: ↑ ***; PT: ↑ ***	↑ *Verrucomicrobia* **, ↑ *Ileibacterium* *, ↑ *Akkermansia* **, ↑ *Blautia* * ↓ *Actinobacteria* *, ↓ *Bifidobacteriales* *, ↓ *Coriobacteriales* ***; ↓ *Dubosiella* *, ↓ *Bifidobacterium* *

Interv.—intervention; MPTP—1-methyl-4-phenyl-1,2,3,6-tetrahydropyridine; 6-OHDA—6-hydroxydopamine; LPS—lipopolysaccharide; ATB—antibiotic; PRO—probiotic; PRE—prebiotic; SYN—synbiotic; FMT—fecal microbiota transplant; SN—substantia nigra; SNpc—substantia nigra pars compacta; STR—striatum; IHC—immunohistochemistry; IF—immunoflorescence; WB—Western Blot, AMPH—amphetamine-induced rotation test; APO—apomorphine-induced rotation test; GA—gait analysis; RR—rotarod; PT—pole test; OF—open field test; BT—narrow beam test; CBT—challenge beam test; CT—cylinder test, TT—traction test; ST—sticker test; EBST—elevated body swing test; GT—grip test; HT—hanging test; AM—FMT from aged mice; YM—FMT from young mice; PD—FMT from Parkinson diseased patients; HC—FMT from healthy controls; Mix—probiotic mixture; 2130—*L. plantarum* CRL 2130; 808—*S. thermophilus* CRL 808; 807—*S. thermophilus* CRL 807; ↑—effect direction increase; ↓—effect direction decrease; * (*p* < 0.05), ** (*p* < 0.01), *** (*p* < 0.001) vs. lesioned/vehicle group; # (*p* < 0.05), ## (*p* < 0.01) vs. lesioned/YM; ns—no statistical significance.

**Table 5:** Tabulation of secondary outcomes for each study included in the systematic review.

Study	Secondary Outcomes
Sun et al., 2018 [78]	Alpha div. NS;
Zhong et al., 2021 [79]	a-syn ↓ ***, TLR4 ↓ Str ***, SN ***; ↓ p-PI3K/PI3K Str ***, SN **, ↓ p-Akt/Akt Str ***, SN ***, NF-κB ↓ Str *, SNpc ***, ↓ AA ***, PA ***, BA ***, N-VA ***
Qiao et al., 2023 [80]	Alpha div. NS; Occludin, Claudin-1 NS, IL-10 Colon NS, SN NS; AM: ↓ BA ^#^ IBA ^###^, IVA ^###^ VA ^##^, PA ^###^, AA ^##^, Colon iNOS NS;
Xie et al., 2023 [81]	PD: GDNF ↑ SNpc ***, Str ***; HC: ↓ SNpc ***, Str ***;
Zhang et al., 2021 [82]	IL-10 ↑ SN *, Colon *, iNOS SN ↓ *
Zhao et al., 2021 [47]	Alpha div.: ↑ Shannon *, ↑ Simpson *; Midbrain a-syn ↓ ***, LPS ↓ SN, Colon ***, Fecal ***, ZO-1 ↑ **, Occludin ↑ ***, Claudin5 ↑ ***, TLR4 ↓ SN ***, Colon ***, COX2 ↓ midbrain *, colon **, SN **; iNOS ↓ midbrain **, colon ***, SN **; NF-κB serum ↓ ***
Lee et al., 2022 [83]	↑ pAKT/AKT **
Castelli et al., 2020 [84]	BDNF ↑ SN ***, Str *; PPaRγ↑ Str ***, SN ***; Pi3K ↑ ***; p-AKT ↑ ***; NF-κB ↓ Str **, SN ***
Liao et al., 2020 [86]	Mature BDNF ↑ *; Alpha div.: ↑ Shannon *, Evenness ***, ↓ Chao1 *; ↓ LPS synthesizing module *, SCFA NS
Perez Visnuk et al., 2020 [87]	Mixture ↑ IL-10 *
Li et al., 2022 [88]	BDNF ↑ Striatum ***; GDNF ↑ Striatum ***; Alpha diversity: ↑ Shannon *, Simpson **; ZO-1 ↓ Striate *, colon *, claudin-1 ↓ Striate *, colon *; ↑ IL-10 colon ***, ↑ AA ***, BA ***, IBA ***, IVA ***
Yue et al., 2022 [89]	BDNF ↑ *; GDNF ↑ *; Alpha diversity: Simpson ↑ *; a-syn ↓ aggregation **, ZO-1 ↓ colon *, occludin ↓ colon *
Sun et al., 2021 [90]	Alpha diversity NS; TLR4 ↓ Str **, Colon *; NF-κB ↓ Str **, Colon *; ↓ AA ***, PA **, BA *, VA **
Pan et al., 2022 [91]	Alpha diversity: ↑ Shannon **, ↓ Simpson **; α-syn ↓ in SN **
Fang et al., 2020 [92]	TLR4↓ *, Akt NS
Srivastav et al., 2019 [93]	MPTP: BDNF ↑ **; GDNF ↑ **; ↑ pPI3K/PI3K *, ↑ pAkt/Akt *
Srivastav et al., 2019 [93]	Rotenone: BDNF ↑ *; GDNF ↑ **;
Lee et al., 2023 [94]	BDNF NS; Alpha diversity NS; IL-10 ↑ SN ***, iNOS ↓
Chu et al., 2023 [95]	Alpha diversity ↓ *; Occludin ↑ *; ZO-1 * ↑
Tsao et al., 2021 [99]	Synbiotic group: Alpha diversity: Shannon ↑ *; All groups ↑ total acid *, PA *, BA *
Liu et al., 2022 [100]	Synbiotic: BDNF ↑ Striatum *; GDNF ↑ Striatum *; Prebiotic: ↑ AA ***, BA ***
Koutzoumis et al., 2019 [41]	↓ COX2 *
Hong et al., 2022 [42]	Alpha diversity ↓ *; ↓ serum occludin * and claudin-5 **
Pu et al., 2019 [101]	Alpha diversity: Shannon ↓ **, Chao1 ↓ ***, ACE ↓ **
Cui et al., 2023 [102]	Alpha diversity: Chao1 ↓ ***, Obs. species index ↑ ***; TLR4 ↓ Str *, colon *; NF-κB ↓ Str *, colon *

MPTP—1-methyl-4-phenyl-1,2,3,6-tetrahydropyridine; SN—substantia nigra; SNpc—substantia nigra pars compacta; Str—striatum; PD—FMT from Parkinson diseased patients; HC—FMT from healthy controls; AM—FMT from aged mice; YM—FMT from young mice; SCFA—short-chain fatty acids; AA—acetic acid; BA—butyric acid; IBA—isobutyric acid; IVA—isovaleric acid; VA—valeric acid; N-VA—N-valeric acid; ↑—effect direction increase; ↓—effect direction decrease; * (*p* < 0.05), ** (*p* < 0.01), *** (*p* < 0.001) lesioned/intervention vs. lesioned/vehicle group; # (*p* < 0.05), ## (*p* < 0.01), ### (*p* < 0.001) AM vs. YM; NS—no statistical difference.

## Data Availability

Data are contained within the article and Supplementary Materials. The protocol of the study was registered in the Prospero database, ID CRD42023461495, https://www.crd.york.ac.uk/prospero/display_record.php?RecordID=461495, accessed on 12 April 2024.

## Supplementary Materials

The following supporting information can be downloaded at: https://www.mdpi.com/article/10.3390/cimb46050244/s1, Table S1: Individual SYRCLE risk of bias; File S1: PRISMA Checklist; File S2: PRISMA Abstracts Checklist; File S3: PRISMA-S Checklist; File S4: SWiM Checklist. Reference [147] is cited in the Supplementary Materials.
