# Supplementary material for: The Effect of Gut Microbiota-Targeted Interventions on Neuroinflammation and Motor Function in Parkinson’s Disease Animal Models—A Systematic Review
Source: Curr Issues Mol Biol. 2024 Apr 26;46(5):3946–74. doi: 10.3390/cimb46050244 (PMC11120577; doi:10.3390/cimb46050244)
Supplement: Supplementary file 1 [file cimb-46-00244-s001.zip › Table S1 - Individual SYRCLE risk of bias.pdf]

Table S1. Individual SYRCLE risk of bias

	SYRCLE risk of bias										
	Selection bias				Performance bias		Detection bias		Attrition bias	Reporting bias	Other
Article Identification	Sequence generation	SPF	Baseline	Allocation concealement	Random housing	Blinding	Random outcome assessment	Blinding	Incomplete outcome data	Selective outcome reporting	Other sourses of bias
Agathobaculum butyriciproducens shows Neuroprotective Effects in a 6-OHDA- Induced Mouse Model of Parkinson's Disease [1]	U	Y	U	U	Y	U	Y	U	Y	Y	N
Alterations of the gut microbiota with antibiotics protects dopamine neuron loss and improve motor deficits in a pharmacological rodent model of Parkinson's disease. [2]	U	N	Y	U	Y	U	U	Y	U	U	N
Chronic Treatment with the Probiotics Lacticaseibacillus rhamnosus GG and Bifidobacterium lactis BB12 Attenuates Motor Impairment, Striatal Microglial Activation, and Dopaminergic Loss in Rats with 6-Hydroxydopamine-induced Hemiparkinsonism. [3]	U	N	U	U	Y	U	Y	Y	Y	Y	N
Dysbiosis of gut microbiota inhibits NMNAT2 to promote neurobehavioral deficits and oxidative stress response in the 6-OHDA-lesioned rat model of Parkinson's disease [4]	U	Y	Y	U	Y	U	Y	U	Y	Y	N
Effects of the probiotic formulation SLAB51 in in vitro and in vivo Parkinson's disease models.[5]	U	N	Y	U	Y	U	U	Y	U	U	N
Fecal Microbiota Transplantation Exerts a Protective Role in MPTP-Induced Parkinson's Disease via the TLR4/PI3K/AKT/NF-kappaB Pathway Stimulated by alpha-Synuclein.[6]	U	N	Y	U	Y	U	Y	U	Y	Y	N

Fecal Microbiota Transplantation from Aged Mice Render Recipient Mice Resistant to MPTP-Induced Nigrostriatal Degeneration Via a Neurogenesis-Dependent but Inflammation-Independent Manner.[7]	U	N	U	U	Y	U	U	U	U	U	N
Fecal microbiota transplantation protects rotenone-induced Parkinson's disease mice via suppressing inflammation mediated by the lipopolysaccharide-TLR4 signaling pathway through the microbiota-gut-brain axis. [8]	U	N	Y	U	Y	U	U	Y	U	U	Y
Gut microbiota relieves inflammation in the substantia nigra of chronic Parkinson's disease by protecting the function of dopamine neurons [9]	U	N	Y	U	U	U	U	U	U	U	Y
Healthy Human Fecal Microbiota Transplantation into Mice Attenuates MPTP-Induced Neurotoxicity via AMPK/SOD2 Pathway [10]	U	N	Y	U	Y	U	U	U	Y	Y	Y
Lactobacillus plantarum CCFM405 against Rotenone-Induced Parkinson’s Disease Mice via Regulating Gut Microbiota and Branched-Chain Amino Acids Biosynthesis [11]	U	N	Y	U	Y	U	Y	Y	Y	N	Y
Lactobacillus plantarum PS128 alleviates neurodegenerative progression in 1-methyl-4-phenyl-1,2,3,6-tetrahydropyridine-induced mouse models of Parkinson's disease [12]	U	Y	Y	U	Y	U	U	Y	Y	Y	Y
Neuroprotective and Immunomodulatory Effects of Probiotics in a Rat Model of Parkinson's Disease. [13]	U	N	Y	U	Y	U	U	U	Y	Y	N
Neuroprotective effects associated with immune modulation by selected lactic acid bacteria in a Parkinson's disease model. [14]	U	Y	Y	U	Y	U	U	U	Y	Y	N
Neuroprotective Effects of Bifidobacterium breve CCFM1067 in MPTP-Induced Mouse Models of Parkinson’s Disease [15]	U	N	U	U	U	U	U	U	U	N	Y
Neuroprotective effects of fecal microbiota transplantation on MPTP-induced Parkinson's disease mice: Gut microbiota, glial reaction and TLR4/TNF-alpha signaling pathway. [16]	U	Y	Y	U	Y	U	U	U	U	U	Y
Neuroprotective Effects of Lactobacillus plantarum PS128 in a Mouse Model of Parkinson’s Disease: The Role of Gut Microbiota and MicroRNAs [17]	U	N	U	U	Y	U	U	U	U	U	Y

Neurotrophic Role of the Next-Generation Probiotic Strain L. lactis MG1363-pMG36e-GLP-1 on Parkinson's Disease via Inhibiting Ferroptosis. [18]	Y	N	U	U	Y	U	U	U	U	U	Y
Polymannuronic acid prebiotic plus Lacticaseibacillus rhamnosus GG probiotic as a novel synbiotic promoted their separate neuroprotection against Parkinson's disease. [19]	U	N	U	U	Y	U	U	U	Y	Y	Y
Probiotic Clostridium butyricum ameliorated motor deficits in a mouse model of Parkinson's disease via gut microbiota-GLP-1 pathway. [20]	U	N	Y	U	U	U	U	U	U	U	Y
Probiotic Enhancement of Antioxidant Capacity and Alterations of Gut Microbiota Composition in 6-Hydroxydopamin-Induced Parkinson's Disease Rats [21]	U	N	Y	U	Y	U	U	U	Y	Y	Y
Probiotic Pediococcus pentosaceus ameliorates MPTP-induced oxidative stress via regulating the gut microbiota-gut-brain axis. [22]	U	N	Y	U	Y	U	U	U	U	N	Y
Probiotics Alleviate the Progressive Deterioration of Motor Functions in a Mouse Model of Parkinson's Disease [23]	U	N	U	U	Y	U	U	U	U	Y	N
Probiotics mixture increases butyrate, and subsequently rescues the nigral dopaminergic neurons from MPTP and rotenone-induced neurotoxicity. [24]	U	N	U	U	Y	U	U	Y	U	U	N
Rifaximin Modifies Gut Microbiota and Attenuates Inflammation in Parkinson's Disease: Preclinical and Clinical Studies. [25]	U	N	U	U	Y	U	U	U	Y	U	Y
Antibiotic-induced microbiome depletion protects against MPTP-induced dopaminergic neurotoxicity in the brain. [26]	U	N	Y	U	Y	U	U	U	U	Y	N
Vancomycin Pretreatment on MPTP-Induced Parkinson's Disease Mice Exerts Neuroprotection by Suppressing Inflammation Both in Brain and Gut. [27]	U	Y	U	U	Y	U	U	U	U	U	Y
The impact of dextran sodium sulphate and probiotic pre-treatment in a murine model of Parkinson's disease [28]	U	N	Y	U	Y	U	U	Y	U	U	Y
Therapeutic effect of GLP-1 engineered strain on mice model of Alzheimer's disease and Parkinson's disease [29]	U	N	Y	U	Y	U	U	U	U	N	N

References

1. Lee, D.W.; Ryu, Y.-K.; Chang, D.-H.; Park, H.-Y.; Go, J.; Maeng, S.-Y.; Hwang, D.Y.; Kim, B.-C.; Lee, C.-H.; Kim, K.-S. Agathobaculum Butyriciproducens Shows Neuroprotective Effects in a 6-OHDA-Induced Mouse Model of Parkinson's Disease. *J. Microbiol. Biotechnol.* **2022**, *32*, 1168–1177, doi:10.4014/jmb.2205.05032.
2. Koutzoumis, D.N.; Vergara, M.; Pino, J.; Buddendorff, J.; Khoshbouei, H.; Mandel, R.J.; Torres, G.E. Alterations of the Gut Microbiota with Antibiotics Protects Dopamine Neuron Loss and Improve Motor Deficits in a Pharmacological Rodent Model of Parkinson's Disease. *Exp. Neurol.* **2020**, *325*, 113159, doi:10.1016/j.expneurol.2019.113159.
3. Cuevas-Carbonell, S.G.; Vásquez-Celaya, L.; García-López, D.; Granados-Patrón, D.; García-Miss, M.D.R.; Álvarez-Cervera, F.J.; Mut-Martín, M.; Parra, I.; Mendieta, L.; Salgado, H.; et al. Chronic Treatment with the Probiotics Lacticaseibacillus Rhamnosus GG and Bifidobacterium Lactis BB12 Attenuates Motor Impairment, Striatal Microglial Activation, and Dopaminergic Loss in Rats with 6-Hydroxydopamine-Induced Hemiparkinsonism. *Neuroscience* **2022**, *507*, 79–98, doi:10.1016/j.neuroscience.2022.11.004.
4. Yu, J.; Meng, J.; Qin, Z.; Yu, Y.; Liang, Y.; Wang, Y.; Min, D. Dysbiosis of Gut Microbiota Inhibits NMNAT2 to Promote Neurobehavioral Deficits and Oxidative Stress Response in the 6-OHDA-Lesioned Rat Model of Parkinson's Disease. *J. Neuroinflammation* **2023**, *20*, 117, doi:10.1186/s12974-023-02782-1.
5. Castelli, V.; d'Angelo, M.; Lombardi, F.; Alfonsetti, M.; Antonosante, A.; Catanesi, M.; Benedetti, E.; Palumbo, P.; Cifone, M.G.; Giordano, A.; et al. Effects of the Probiotic Formulation SLAB51 in in Vitro and in Vivo Parkinson's Disease Models. *Aging* **2020**, *12*, 4641–4659, doi:10.18632/aging.102927.
6. Zhong, Z.; Chen, W.; Gao, H.; Che, N.; Xu, M.; Yang, L.; Zhang, Y.; Ye, M. Fecal Microbiota Transplantation Exerts a Protective Role in MPTP-Induced Parkinson's Disease via the TLR4/PI3K/AKT/NF-κB Pathway Stimulated by α-Synuclein. *Neurochem. Res.* **2021**, *46*, 3050–3058, doi:10.1007/s11064-021-03411-0.
7. Qiao, C.-M.; Zhou, Y.; Quan, W.; Ma, X.-Y.; Zhao, L.-P.; Shi, Y.; Hong, H.; Wu, J.; Niu, G.-Y.; Chen, Y.-N.; et al. Fecal Microbiota Transplantation from Aged Mice Render Recipient Mice Resistant to MPTP-Induced Nigrostriatal Degeneration Via a Neurogenesis-Dependent but Inflammation-Independent Manner. *Neurother. J. Am. Soc. Exp. Neurother.* **2023**, *20*, 1405–1426, doi:10.1007/s13311-023-01420-1.
8. Zhao, Z.; Ning, J.; Bao, X.-Q.; Shang, M.; Ma, J.; Li, G.; Zhang, D. Fecal Microbiota Transplantation Protects Rotenone-Induced Parkinson's Disease Mice via Suppressing Inflammation Mediated by the Lipopolysaccharide-TLR4 Signaling Pathway through the Microbiota-Gut-Brain Axis. *Microbiome* **2021**, *9*, 226, doi:10.1186/s40168-021-01107-9.
9. Zhang, T.; Wang, T.; Chen, X.; Zhao, Z.; Chen, Z. Gut Microbiota Relieves Inflammation in the Substantia Nigra of Chronic Parkinson's Disease by Protecting the Function of Dopamine Neurons. *Exp. Ther. Med.* **2022**, *23*, 52, doi:10.3892/etm.2021.10974.
10. Xie, Z.; Zhang, M.; Luo, Y.; Jin, D.; Guo, X.; Yang, W.; Zheng, J.; Zhang, H.; Zhang, L.; Deng, C.; et al. Healthy Human Fecal Microbiota Transplantation into Mice Attenuates MPTP-Induced Neurotoxicity via AMPK/SOD2 Pathway. *Aging Dis.* **2023**, *14*, 2193–2214, doi:10.14336/AD.2023.0309.
11. Chu, C.; Yu, L.; Li, Y.; Guo, H.; Zhai, Q.; Chen, W.; Tian, F. Lactobacillus Plantarum CCFM405 against Rotenone-Induced Parkinson's Disease Mice via Regulating Gut Microbiota and Branched-Chain Amino Acids Biosynthesis. *Nutrients* **2023**, *15*, 1737, doi:10.3390/nu15071737.
12. Liao, J.-F.; Cheng, Y.-F.; You, S.-T.; Kuo, W.-C.; Huang, C.-W.; Chiou, J.-J.; Hsu, C.-C.; Hsieh-Li, H.-M.; Wang, S.; Tsai, Y.-C. Lactobacillus Plantarum PS128 Alleviates Neurodegenerative Progression in 1-Methyl-4-Phenyl-1,2,3,6-Tetrahydropyridine-Induced Mouse Models of Parkinson's Disease. *Brain. Behav. Immun.* **2020**, *90*, 26–46, doi:10.1016/j.bbi.2020.07.036.
13. Parra, I.; Martínez, I.; Vásquez-Celaya, L.; Gongora-Alfaro, J.L.; Tizabi, Y.; Mendieta, L. Neuroprotective and Immunomodulatory Effects of Probiotics in a Rat Model of Parkinson's Disease. *Neurotox. Res.* **2023**, *41*, 187–200, doi:10.1007/s12640-022-00627-y.
14. Perez Visñuk, D.; Savoy de Giori, G.; LeBlanc, J.G.; de Moreno de LeBlanc, A. Neuroprotective Effects Associated with Immune Modulation by Selected Lactic Acid Bacteria in a Parkinson's Disease Model. *Nutr. Burbank Los Angel. Cty. Calif* **2020**, *79–80*, 110995, doi:10.1016/j.nut.2020.110995.
15. Li, T.; Chu, C.; Yu, L.; Zhai, Q.; Wang, S.; Zhao, J.; Zhang, H.; Chen, W.; Tian, F. Neuroprotective Effects of Bifidobacterium Breve CCFM1067 in MPTP-Induced Mouse Models of Parkinson's Disease. *Nutrients* **2022**, *14*, 4678, doi:10.3390/nu14214678.

16. Sun, M.-F.; Zhu, Y.-L.; Zhou, Z.-L.; Jia, X.-B.; Xu, Y.-D.; Yang, Q.; Cui, C.; Shen, Y.-Q. Neuroprotective Effects of Fecal Microbiota Transplantation on MPTP-Induced Parkinson's Disease Mice: Gut Microbiota, Glial Reaction and TLR4/TNF-α Signaling Pathway. *Brain. Behav. Immun.* **2018**, *70*, 48–60, doi:10.1016/j.bbi.2018.02.005.
17. Lee, Y.Z.; Cheng, S.-H.; Chang, M.-Y.; Lin, Y.-F.; Wu, C.-C.; Tsai, Y.-C. Neuroprotective Effects of Lactobacillus Plantarum PS128 in a Mouse Model of Parkinson's Disease: The Role of Gut Microbiota and MicroRNAs. *Int. J. Mol. Sci.* **2023**, *24*, 6794, doi:10.3390/ijms24076794.
18. Yue, M.; Wei, J.; Chen, W.; Hong, D.; Chen, T.; Fang, X. Neurotrophic Role of the Next-Generation Probiotic Strain L. Lactis MG1363-pMG36e-GLP-1 on Parkinson's Disease via Inhibiting Ferroptosis. *Nutrients* **2022**, *14*, 4886, doi:10.3390/nu14224886.
19. Liu, X.; Du, Z.R.; Wang, X.; Sun, X.R.; Zhao, Q.; Zhao, F.; Wong, W.T.; Wong, K.H.; Dong, X.-L. Polymannuronic Acid Prebiotic plus Lacticaseibacillus Rhamnosus GG Probiotic as a Novel Synbiotic Promoted Their Separate Neuroprotection against Parkinson's Disease. *Food Res. Int. Ott. Ont* **2022**, *155*, 111067, doi:10.1016/j.foodres.2022.111067.
20. Sun, J.; Li, H.; Jin, Y.; Yu, J.; Mao, S.; Su, K.-P.; Ling, Z.; Liu, J. Probiotic Clostridium Butyricum Ameliorated Motor Deficits in a Mouse Model of Parkinson's Disease via Gut Microbiota-GLP-1 Pathway. *Brain. Behav. Immun.* **2021**, *91*, 703–715, doi:10.1016/j.bbi.2020.10.014.
21. Tsao, S.-P.; Nurrahma, B.A.; Kumar, R.; Wu, C.-H.; Yeh, T.-H.; Chiu, C.-C.; Lee, Y.-P.; Liao, Y.-C.; Huang, C.-H.; Yeh, Y.-T.; et al. Probiotic Enhancement of Antioxidant Capacity and Alterations of Gut Microbiota Composition in 6-Hydroxydopamin-Induced Parkinson's Disease Rats. *Antioxid. Basel Switz.* **2021**, *10*, 1823, doi:10.3390/antiox10111823.
22. Pan, S.; Wei, H.; Yuan, S.; Kong, Y.; Yang, H.; Zhang, Y.; Cui, X.; Chen, W.; Liu, J.; Zhang, Y. Probiotic Pediococcus Pentosaceus Ameliorates MPTP-Induced Oxidative Stress via Regulating the Gut Microbiota-Gut-Brain Axis. *Front. Cell. Infect. Microbiol.* **2022**, *12*, 1022879, doi:10.3389/fcimb.2022.1022879.
23. Hsieh, T.-H.; Kuo, C.-W.; Hsieh, K.-H.; Shieh, M.-J.; Peng, C.-W.; Chen, Y.-C.; Chang, Y.-L.; Huang, Y.-Z.; Chen, C.-C.; Chang, P.-K.; et al. Probiotics Alleviate the Progressive Deterioration of Motor Functions in a Mouse Model of Parkinson's Disease. *Brain Sci.* **2020**, *10*, 206, doi:10.3390/brainsci10040206.
24. Srivastav, S.; Neupane, S.; Bhurtel, S.; Katila, N.; Maharjan, S.; Choi, H.; Hong, J.T.; Choi, D.-Y. Probiotics Mixture Increases Butyrate, and Subsequently Rescues the Nigral Dopaminergic Neurons from MPTP and Rotenone-Induced Neurotoxicity. *J. Nutr. Biochem.* **2019**, *69*, 73–86, doi:10.1016/j.jnutbio.2019.03.021.
25. Hong, C.-T.; Chan, L.; Chen, K.-Y.; Lee, H.-H.; Huang, L.-K.; Yang, Y.-C.S.H.; Liu, Y.-R.; Hu, C.-J. Rifaximin Modifies Gut Microbiota and Attenuates Inflammation in Parkinson's Disease: Preclinical and Clinical Studies. *Cells* **2022**, *11*, 3468, doi:10.3390/cells11213468.
26. Pu, Y.; Chang, L.; Qu, Y.; Wang, S.; Zhang, K.; Hashimoto, K. Antibiotic-Induced Microbiome Depletion Protects against MPTP-Induced Dopaminergic Neurotoxicity in the Brain. *Aging* **2019**, *11*, 6915–6929, doi:10.18632/aging.102221.
27. Cui, C.; Hong, H.; Shi, Y.; Zhou, Y.; Qiao, C.-M.; Zhao, W.-J.; Zhao, L.-P.; Wu, J.; Quan, W.; Niu, G.-Y.; et al. Vancomycin Pretreatment on MPTP-Induced Parkinson's Disease Mice Exerts Neuroprotection by Suppressing Inflammation Both in Brain and Gut. *J. Neuroimmune Pharmacol. Off. J. Soc. NeuroImmune Pharmacol.* **2023**, *18*, 72–89, doi:10.1007/s11481-021-10047-y.
28. Dwyer, Z.; Chaiquin, M.; Landrigan, J.; Ayoub, K.; Shail, P.; Rocha, J.; Childers, C.L.; Storey, K.B.; Philpott, D.J.; Sun, H.; et al. The Impact of Dextran Sodium Sulphate and Probiotic Pre-Treatment in a Murine Model of Parkinson's Disease. *J. Neuroinflammation* **2021**, *18*, 20, doi:10.1186/s12974-020-02062-2.
29. Fang, X.; Zhou, X.; Miao, Y.; Han, Y.; Wei, J.; Chen, T. Therapeutic Effect of GLP-1 Engineered Strain on Mice Model of Alzheimer's Disease and Parkinson's Disease. *AMB Express* **2020**, *10*, 80, doi:10.1186/s13568-020-01014-6.
